# Changes in the Cytoplasmic Composition of Amino Acids and Proteins Observed in *Staphylococcus aureus* during Growth under Variable Growth Conditions Representative of the Human Wound Site

**DOI:** 10.1371/journal.pone.0159662

**Published:** 2016-07-21

**Authors:** Mousa M. Alreshidi, R. Hugh Dunstan, Johan Gottfries, Margaret M. Macdonald, Marcus J. Crompton, Ching-Seng Ang, Nicholas A. Williamson, Tim K. Roberts

**Affiliations:** 1 Metabolic Research Group, Faculty of Science and Information Technology, School of Environmental and Life Sciences, Department of Biology, University Drive, Callaghan, 2308, NSW, Australia; 2 Department of Chemistry and Molecular Biology, Gothenburg University, Sweden; 3 Bio21 Molecular Science and Biotechnology Institute, University of Melbourne, Victoria 3010, Australia; 4 Department of Biology, College of Science, University of Ha’il, P.O. 2440, Hail, Saudi Arabia; National Institute of Agronomic Research, FRANCE

## Abstract

*Staphylococcus aureus* is an opportunistic pathogen responsible for a high proportion of nosocomial infections. This study was conducted to assess the bacterial responses in the cytoplasmic composition of amino acids and ribosomal proteins under various environmental conditions designed to mimic those on the human skin or within a wound site: pH6-8, temperature 35–37°C, and additional 0–5% NaCl. It was found that each set of environmental conditions elicited substantial adjustments in cytoplasmic levels of glutamic acid, aspartic acid, proline, alanine and glycine (P< 0.05). These alterations generated characteristic amino acid profiles assessed by principle component analysis (PCA). Substantial alterations in cytoplasmic amino acid and protein composition occurred during growth under conditions of higher salinity stress implemented via additional levels of NaCl in the growth medium. The cells responded to additional NaCl at pH 6 by reducing levels of ribosomal proteins, whereas at pH 8 there was an upregulation of ribosomal proteins compared with the reference control. The levels of two ribosomal proteins, L32 and S19, remained constant across all experimental conditions. The data supported the hypothesis that the bacterium was continually responding to the dynamic environment by modifying the proteome and optimising metabolic homeostasis.

## Introduction

*Staphylococcus aureus* is ubiquitous in the environment where it can be found on skin surfaces and body orifices, as well as in the water, air, food and dust [1]. It can also be found on inanimate surfaces including medical instruments and therefore has been identified as the major cause of infections associated with many surgical procedures including insertion of artificial heart valves, prosthetic joints and cerebrospinal fluid shunts [2, 3]. *S*. *aureus* is routinely exposed to a dynamic environment where the bacteria must survive on fomites, organic debris, food and water in transition between hosts and then come into contact with potential new hosts via deposition onto skin surfaces, ingestion within food or direct insertion into wound sites. Once they have entered a wound site, the bacteria need to establish in sufficient numbers and counter the host defence systems during the infection process. The wound site represents an excellent example of a stable yet dynamic environment where the pH level can be either neutral or acidic during the infection but increases from 7.5–8.9 during the healing process. Concentrations of NaCl can also increase in response to traumatization [4] and, as the tissue is repaired, the pH becomes more acidic and can reach a pH of 5.5 [5]. The skin temperature can be 2–4°C lower than the base level temperature depending on the surrounding conditions and the location of the wound on the body [6], but it can also increase by 2–4°C as a result of localised inflammation [7]. It has been found that staphylococcal species were the most common group of bacteria identified in the healing and non-healing wound sites but *S*. *aureus* was the most prevalent bacterium in the non-healing wound sites [8, 9]. This observation suggested that this bacterium had a role in prolonging or stopping the healing processes.

The adaptation mechanisms required for surviving dynamic environments involve continuous adjustments of metabolic homeostasis with concomitant genomic, proteomic and structural modifications within the cell to help cope with changes in pH, temperature, salinity pressure, nutrient availability and exposure to toxic chemicals. These responses and adjustments in metabolism lead to the formation of heterogeneity within the bacterial population to maximise the chances of survival [10]. One of the best examples of heterogeneity is the formation of small colony variants (SCVs) that can occur following exposure to antibiotics [10] or environmental stressors such as low temperature [11]. SCV formation is a common mechanism of phenotypic shift used by staphylococcal species in response to toxic chemical or environmental stressors resulting in a more resilient phenotype which enhances survival of these species [11–14]. This phenotypic conversion coupled with the enhanced capacity for biofilm formation offer defence mechanisms against both the host immune system and harsh environments [13, 14]. These phenomena were thought to be due to the ability of the bacterium to alter its metabolism and protein production [15, 16]. The exposure of *S*. *aureus* to cold stress has led to substantial alterations in cytoplasmic amino acid composition and proteomic profiles [17]. The prolonged exposure to 4°C resulted in the increased abundance of nine ribosomal proteins whilst the other ribosomal proteins did not alter in abundance relative to reference control samples. This result suggested that certain ribosomal proteins may have specific extra-ribosomal roles in survival under harsh conditions which may include performing as regulatory proteins or forming complexes with other cellular constituents [18].

Evidence has emerged that the exposure of *S*. *lugdunensis* to combinations of changes in pH, temperature and salinity pressure which mimic the ranges found in the wound site led to significant changes in the membrane fatty acid composition, cell size and antibiotic resistance [19]. Another study applied the same experimental regime to monitor the phenotypic shifting of *S*. *aureus* using fourier transform infrared spectroscopy (FTIR) [20] which provided evidence that *S*. *aureus* had a unique phenotype under each condition. It was hypothesised that the facilitation of these changes in the cell size and phenotype would require changes in the metabolic and proteomic profiles and would specifically involve extra-ribosomal functions of certain ribosomal proteins. Therefore, an experimental scheme was designed in this study to investigate how the cytoplasmic composition of amino acids and ribosomal proteins in *S*. *aureus* would alter in response to changes in the environmental conditions designed to mimic those on the human skin or within a wound site: pH6-8, temperature 35–37°C, and additional 0–5% NaCl. To assess changes in metabolic homeostasis after growth under the various regimes, cells were harvested to measure 1) amino acid concentrations in the cytoplasm, and 2) variations in relative abundances of ribosomal proteins. It was hypothesised that each set of environmental conditions would lead to significant alterations in the amino acids and ribosomal proteins of *S*. *aureus*. These data would allow insight into how *S*. *aureus* adapts rapidly to environmental stimuli which in turn could help identify new target areas of metabolic control for the development of novel antimicrobial strategies.

## Materials and Methods

The bacterial strain used in this study was a clinical isolate of *S*. *aureus* from patients that had been suffering from chronic muscle pain [21]. This isolate has been used in subsequent investigations to investigate metabolic responses to environmental stresses [11, 13]. The isolate has been maintained as culture stock on horse blood agar (HBA) and preserved appropriately on sterile glass beads at -80°C with a regular sub-culturing to maintain viability. The identity of the isolate was checked regularly using API^™^ Staph biochemistry and through PCR [22].

The bacteria were grown under the range of conditions described in Crompton *et al* 2014 as part of a larger experiment designed by MODDE software (9.0, Umetrics Sweden) which allows the establishment of a multifactorial design optimised to assess the impact of varying three factors at a time, which in this case were temperature, pH and salinity. Due to logistical constraints, a subset of environmental conditions was selected for analyses of both metabolic composition as well as proteomic composition in the bacteria following growth. The reference control included cells grown under ideal conditions of pH7 at 37°C with no added NaCl in tryptic soy broth medium (TSB); a “centroid” set of samples represented the mid-range conditions of the larger experimental design (Crompton *et al* 2014) with conditions of pH7 at 37°C with NaCl added (2.5%) in TSB; four sets (each n = 4) of experimental conditions were applied with 1) 35°C and pH6 with no added NaCl in TSB; 2) 35°C and pH6 with NaCl added (5%) in TSB; 3) 35°C and pH8 with no added NaCl in TSB; 4) 35°C and pH8 with NaCl added (5%) in TSB. The TSB growth medium had a sodium concentration of 85mM compared with a range of 135-145mM noted for plasma. However the osmolarity of the TSB was measured as 300 mosmoles L^-1^ and the calculated osmolarity of PBS was 306 mosmoles L^-1^ [23]. The sodium chloride ranged from 0 to an additional 5% loading on the TSB, taking the final concentration well beyond the plasma concentration which may occur on skin surfaces with added electrolytes from the natural moisturising factor and sweat [24] and has also been noted in wound site responses to traumatization [4]. The temperature range was 35–37°C and the pH ranged from 6–8. The sets of control (n = 4) and centroid (n = 4) samples were repeated 3 separate times across the period of experimentation to control for temporal variations (giving final replicate numbers of n = 12 for these sets). One or more of the four sets of experimental conditions were performed at the same time as the control and centroid samples. An overnight starter culture (50 ml) of *S*. *aureus* was grown for 16h in Tryptic Soy Broth (TSB) at 37°C with constant agitation (120 rpm) to be used as an inoculum for the growth experiments. Replicates of each condition containing 95 ml TSB culture media were inoculated with 5 ml of overnight culture in 500 ml conical flasks which were then grown until mid-exponential phase with constant agitation (120 rpm). Harvested cells were washed three times using phosphate buffered saline (PBS) at 4°C. In a separate experiment, cells were incubated in PBS with glucose and glycine (osmolarity 306 mosmoles L^-1^) for 30 minutes and then washed three times with either isotonic PBS or Millipore Milli-Q H_2_O. The washings were analysed for evidence of cell lysis via the release of glutamic acid and other amino acids. No cytoplasmic amino acids were found in the washing solutions verifying that cell lysis or “downshock” release of amino acids did not occur [25]. The washed cells were immediately quenched using liquid nitrogen for lyophilisation and subsequent extraction for metabolomic and proteomic analysis.

Proteins were extracted from the cytoplasm of dried cells from both the reference control and the experimental samples. The cells were resuspended in 500 μl of SDS Lysis Buffer containing 2% SDS, 0.375 M Tris pH 6.8, 3.4 M sucrose (Sigma-Aldrich) and 1 tablet of protease inhibitor (complete Mini, Roche Diagnostics) was added. The re-suspension mixture was thoroughly mixed and sonicated for 5 min using a water bath sonicator. Samples were then heated at 100°C in a dry heating block for 6 min and cell debris was removed by centrifugation at 14,000 rpm (Eppendorf Microcentrifuge 5418) for 25 min. The supernatant containing the extracted proteins was carefully removed and stored at –20°C until further investigation. Protein concentrations in each replicate of the six sets of control (n = 12), centroid (n = 12) and experimental (n = 4 each) conditions were determined using the BCA^™^ assay (Bio-Rad) following the manufacturer’s instructions and Bovine Serum Albumin (BSA) was used as the reference standard.

Aliquots from each sample containing 100 μg proteins were precipitated using sample/methanol/chloroform in the ratio of 1:1:0.5 (v/v/v). The mixture was vortexed and centrifuged at 14,000 rpm for 15min. The upper layer was discarded and 75% of the original volume of methanol was added to each replicate and centrifuged at 14,000 rpm for 20 min. The supernatant was removed without disrupting the protein pellet which was then air dried for 10 min. The dried protein pellet was washed twice with 500μL of cold acetone (kept at -20°C) and then centrifuged at 14,000 rpm for 20 min. Precipitated proteins were resuspended with 150 μl of ammonium bicarbonate (25 mM) and 10mM dithiothreitol (DTT, final concentration) and boiled at 95°C for 10 min. The samples were cooled for 5 min and iodacetamide subsequently added to give a final concentration of 55mM. The samples were then vortexed and kept in the dark at room temperature for 35 min. Protein was digested using trypsin enzyme (Promega sequence grade) at a ratio of 50:1 (protein: trypsin) in 25 mM ammonium bicarbonate and incubated overnight at 37°C with constant shaking. Trypsin digestion was inactivated by adding formic acid to final 1% v/v before MS analysis.

The peptides yielded from the tryptic digestion of cytoplasmic proteins were analysed by nanoLC-MS/MS using a LTQ Orbitrap Elite mass spectrometer (Thermo Scientific) coupled to an Ultimate 3000 RSLC nanosystem (Dionex). The nanoLC system was equipped with a Acclaim Pepmap nano-trap column (Dionex—C18, 100 Å, 75 μm x 2 cm) and a Thermo EASY-Spray column (Pepmap RSLC C18, 2μm, 100 Å, 75 μm x 25 cm). Four μl of the peptide mix was loaded onto the enrichment (trap) column at an isocratic flow of 5 μl/min of 3% CH_3_CN containing 0.1% formic acid for 5 min before the enrichment column was switched in-line with the analytical column. The eluents used for the liquid chromatography were 0.1% (v/v) formic acid (solvent A) and 100% CH_3_CN/0.1% formic acid (v/v). The following flow gradient was used: 3% to 6% B for 1 min, 6% to 10% B in 12 min, 10% to 30% B in 20 min, 30% to 45% B in 2min, 45% to 80% B in 2 min and maintained at 80% B for 3 min followed by equilibration at 3% B for 7min before the next sample injection. The LTQ Orbitrap Elite mass spectrometer was operated in the data dependent mode with nano ESI spray voltage of +2.0 kv, capillary temperature of 250°C and S-lens RF value of 60%.

A data dependent mode was utilised whereby spectra were acquired first in positive mode with full scan scanning from m/z 300–1650 in the FT mode at 120,000 resolution followed by Collision induced dissociation (CID) in the linear ion trap with the ten most intense peptide ions with charge states ≥2 isolated and fragmented using normalized collision energy of 35 and activation Q of 0.25.

The mass spectra were searched using Mascot 2.3 (Matrix Science) as part of the Proteome Discoverer 1.4 Workflow (Thermo Scientific) against the Uniprot database (with 26,167,536 sequences at time of search) and the Firmicutes taxonomy. Search parameters used were: fixed modification (carbamylation of cysteine C; 57), variable modification (oxidation of methionine M; 16), 2 missed tryptic cleavages, 10 ppm peptide mass tolerance and 0.6 Da fragment ion mass tolerance. The false-discovery rate (derived from corresponding decoy database search) was less than 1%.

The MS result files were imported to SIEVE (Version2.1 Thermo Scientific, San Jose, CA, USA) for label free relative quantification of peptides between reference control and treated samples. The experiment workflow was selected as “Control Compare Trend Analysis” which allows the comparison of control samples to more than one treated sample. There are three main steps that include: firstly, alignment of all peptides which considers the reproducibility of replicates and the correlation of all files to a reference file; secondly, the creation of frames (Peptides) and the following parameters were formatted to create the frames (10,000 frames, with signal threshold > 125,000, m/z starts at 350 and stop at 1,300 and retention time (RT) starts = 10 and stops = 60 min) [26]; thirdly the identification of protein and this was done by importing the Mascot 2.1 result to SIEVE 2.1. A filter was applied to the frames table and subsequently the peptides table.

The Frame data generated by SEIVE was exported to an Excel^®^ (Microsoft^®^) spreadsheet file for further data analysis using ANOVA analysis, Principal Component Analysis (PCA) (SIMCA- Software).

Cytoplasmic metabolites were extracted from approximately 10–12 mg of lyophilised cells from each of the replicates. The freeze-dried material was resuspended with 10 ml of 1:1(v/v) cold methanol/water at -20°C and then mixed thoroughly. The methanol/water lyophilised cell slurries were snap-frozen in liquid nitrogen and placed at -20°C for 30 min for a process of slow thawing. Metabolites were separated from the cell debris by centrifugation at 6,500 x g for 25 min. The supernatants containing the metabolites were dried using a vacuum concentrator (CentriVap, LABCORNCO, VWR) and the dried metabolites were subsequently redissolved in 500 μl of sterile Milli-Q water. This process optimised cell extraction whilst minimising processing artefacts on amino acid composition [17, 25, 27].

The amino acid metabolites were analysed using a commercial analytical kit (Phenomenex^®^ EZ: faast^™^). The method was conducted according to the manufacturer’s instructions. The derivatised amino acids were then separated by an Agilent gas chromatograph (Hewlett Packard HP 6890 series) coupled with a flame ionization detector which was calibrated to measure more than 40 amino acid metabolites as previously described [11]. The injection volume was 2 μl with splitless mode and flow rate of the carrier gas (Helium) was 0.5 ml/min. Norvaline was used as an internal standard to calculate the concentrations of amino acids present in the sample as nmol/mg cell dry weight.

The acquired amino acid data obtained from GC-FID were imported to STATISTICA 6 where ANOVA was performed to find the amino acids that were significantly altered after being exposed to stress. Principal Component Analysis (PCA) was then performed utilizing SIMCA-p+ (13.0, Umetrics Sweden)[11, 19, 28]. The data were subjected to mean centring and unit variance (UV) scaling before PCA calculations. The model complexity and validity were assessed by cross-validation (CV) as implemented in the software, i.e. leave one 1/7^th^ of data by the jackknifing procedure [29].

## Results

The clinical strain of *S*. *aureus* was grown in TSB to the mid-exponential phase of growth under various sets of conditions that were representative of the environmental parameters operating within a wound site, with temperatures 35–37°C, pH 6–8 and elevated NaCl (0–5%) above plasma levels. The cytoplasmic amino acid profiles measured in each of the replicates were consistent and reproducible within each of the treatment regimens with specific alterations in composition associated with each treatment (Table 1). Within the range of conditions, a reference set of control samples was defined that represented optimal growing conditions for *S*. *aureus* at 37°C, pH 7 and no added NaCl. Analyses of the cytoplasmic amino acids from the control samples revealed that glutamic acid and aspartic acid were the major cytoplasmic amino acids representing 42% and 29% of the amino acid pool respectively (Table 1). The total abundance of amino acids under control conditions was 215 ± 25 nmoles mg^-1^ (mean ± SD), but the addition of 2.5% NaCl in the “centroid” samples resulted in a significant reduction in cytoplasmic amino acids to 173 ± 19 nmoles mg^-1^. This was primarily achieved by 30–40% decreases in glutamic acid and aspartic acid with a concurrent increase in proline from 7% to 22% of the cytoplasmic amino acid pool. Several significant alterations in other amino acids were also noted as shown in Table 1. Exposures of cells to growth at pH6 at 35°C and no added NaCl resulted in a similar reduction of total amino acids in the cytoplasm compared with the control, but this was achieved by less severe reductions in glutamic and aspartic acids as well as a decrease in proline relative to the control.

**Table 1:** The concentrations of *S*. *aureus* cytoplasmic amino acids following growth under differing experimental regimes.

Treatments	(A) Control pH7; 37°C; 0% NaCl nmoles mg^-1^ dry cell mass; mean ± SD, n = 12	(B) Centroid pH7; 37°C; 2.5% NaCl nmoles mg^-1^ dry cell mass; mean ± SD, n = 12	(C) pH6; 35°C; 0% NaCl nmoles mg^-1^ dry cell mass mean ± SD, n = 4	(D) pH8; 35°C; 0% NaCl nmoles mg^-1^ dry cell mass mean ± SD, n = 4	(E) pH6; 35°C; 5% NaCl nmoles mg^-1^ dry cell mass mean ± SD, n = 4	(F) pH8; 35°C; 5% NaCl nmoles mg^-1^ dry cell mass mean ± SD, n = 4
Amino acids						
**ALA**	13.8 ± 2.77	10.7 ± 3.52	12.4 ± 0.14	15.4 ± 1.98	29.5 ± 2.49^a^	17.1 ± 2.00 ^a^
**GLY**	1.14± 0.29	1.21± 0.51	0.76± 0.01	1.35± 0.17	2.48± 0.74 ^a^	4.89± 0.56 ^a^
**ABA**	1.95 ±0.50	1.75 ±0.14	0.78 ±0.02 ^a^	3.72 ±0.48 ^a^	1.93 ±0.11	4.37 ±0.55 ^a^
**VAL**	1.66 ± 0.82	0.64 ± 0.31 ^a^	1.36 ± 0.79 ^a^	3.26 ± 0.40	1.52 ± 0.16	1.59 ± 0.14
**BAIB**	7.47 ± 1.10	4.24 ± 1.11 ^a^	4.66 ± 0.31 ^a^	7.06 ± 1.01	0.71 ± 0.05 ^a^	2.86 ± 0.07 ^a^
**LEU**	0.81 ± 0.62	0.42 ± 0.07	0.42 ± 0.03	4.76 ± 0.62 ^a^	0.08 ± 0.10 ^a^	0.40 ± 0.14
**ILE**	0.16 ± 0.14	0.07 ± 0.10	0.23 ± 0.16	0.46 ± 0.06 ^a^	0.44 ± 0.05 ^a^	0.28 ± 0.03
**SER**	0.14 ± 0.33	0.24 ± 0.44	0.00 ± 0.00	0.00 ± 0.00	0.29 ± 0.34	0.00 ± 0.00
**PRO**	15.5 ± 3.71	37.6 ± 2.43 ^a^	7.39 ± 0.82 ^a^	28.4 ± 3.51 ^a^	26.9 ± 1.66 ^a^	7.13 ± 1.04 ^a^
**ASN**	0.74 ± 0.17	0.72 ± 0.40	0.09 ± 0.17 ^a^	2.56 ± 0.41 ^a^	2.63 ± 0.12 ^a^	3.93 ± 0.54 ^a^
**ASP**	62.9 ± 8.14	38.3 ± 4.11 ^a^	45.7 ± 6.41 ^a^	91.6 ± 15.91 ^a^	41.1 ± 3.99 ^a^	45.3 ± 2.19 ^a^
**MET**	2.64 ± 0.57	1.99 ± 0.60	2.57 ± 0.34	3.28 ± 0.40 ^a^	1.70 ± 0.14 ^a^	2.54 ± 0.19
**HYP**	0.25 ± 0.19	0.34 ± 0.17	0.63 ± 0.08 ^a^	1.02 ± 0.16 ^a^	0.96 ± 0.15 ^a^	0.69 ± 0.10 ^a^
**GLU**	91.3 ± 11.65	66.5 ± 10.56 ^a^	80.4 ± 10.40	130.2 ± 20.55 ^a^	122.8 ± 13.09 ^a^	95.7 ± 11.09
**PHE**	0.88 ± 0.23	0.50 ± 0.06	0.94 ± 0.11 ^a^	1.73 ± 0.20 ^a^	0.45 ± 0.08 ^a^	0.55 ± 0.13 ^a^
**AAA**	0.32 ± 0.29	0.22 ± 0.31	0.80 ± 0.73 ^a^	0.97 ± 0.12 ^a^	0.00 ± 0.00	0.00 ± 0.00
**GLN**	0.71 ± 0.35	0.52 ± 0.47	1.98 ± 2.56 ^a^	1.37 ± 0.91	5.17 ± 0.50 ^a^	0.67 ± 0.22
**ORN**	0.60 ± 0.10	0.49 ± 0.10	0.90 ± 0.11 ^a^	0.66 ± 0.10	0.86 ± 0.04 ^a^	0.70 ± 0.13
**GPR**	0.77 ± 0.17	0.88 ± 0.17	0.55 ± 0.37	0.79 ± 0.10	0.00 ± 0.00 ^a^	0.20 ± 0.13 ^a^
**LYS**	3.26 ± 1.13	2.29 ± 0.45 ^a^	5.29 ± 0.71 ^a^	4.07 ± 0.53	3.57 ± 0.45	2.41 ± 0.21
**HIS**	7.54 ± 2.65	2.92 ± 1.55 ^a^	5.21 ± 2.59	5.11 ± 3.52	4.28 ± 0.38 ^a^	2.26 ± 0.39 ^a^
**TYR**	0.40 ± 0.08	0.35 ± 0.04	0.47 ± 0.06	0.60 ± 0.05 ^a^	0.33 ± 0.04	0.35 ± 0.07
**Totals**	215±25.2	173±19.3 ^a^	174±19.2 ^a^	308±48.1 ^a^	248±19.2	194±15.7

Cells were harvested and extracted at the mid-exponential phase of growth: (A) control cultures grown under ideal conditions at pH7 and 37°C with no added NaCl; (B) centroid cultures grown at pH 7 and 37°C with 2.5% NaCl, and the four experimental groups: (C) 35°C and pH6 with no added NaCl; (D) 35°C and pH8 with no added NaCl; (E) 35°C and pH6 with 5% NaCl added; (F) 35°C and pH8 with 5% NaCl.

^a^ Significantly different at P<0.05.

Exposing the cells to the lower temperature of 35°C with no added salt at pH 8 had a remarkably different effect to cytoplasmic amino acid composition compared with those observed at pH6. At pH 8, these cells had a 44% increase in cytoplasmic amino acid concentrations achieved primarily by substantial elevations in glutamic acid, aspartic acid, proline and leucine. The addition of 5% NaCl at pH6 and 35°C led to another altered outcome, with increased cytoplasmic amino acids characterized by elevated levels of glutamic acid, proline, alanine and glycine, whilst the concentration of aspartic acid was reduced relative to the control. In contrast, altering the conditions to pH 8 at 35°C and 5% added NaCl led to no significant change in total amino acids relative to the control, but changes in the relative abundances were noted in comparison with the control with increases in glutamic acid, asparagine, α-aminobutyric acid, alanine and glycine as well as decreases in aspartic acid, proline, histidine and β-amino-isobutyric acid. It was thus found that the cytoplasmic amino acid composition profiles were significantly altered in response to growth under the different sets of environmental parameters with alterations in glutamic acid, aspartic acid, proline and alanine representing the major changes in abundance. The results indicated that the cytoplasmic responses to the addition of NaCl differed depending on the prevailing pH and temperature.

**Fig 1:**
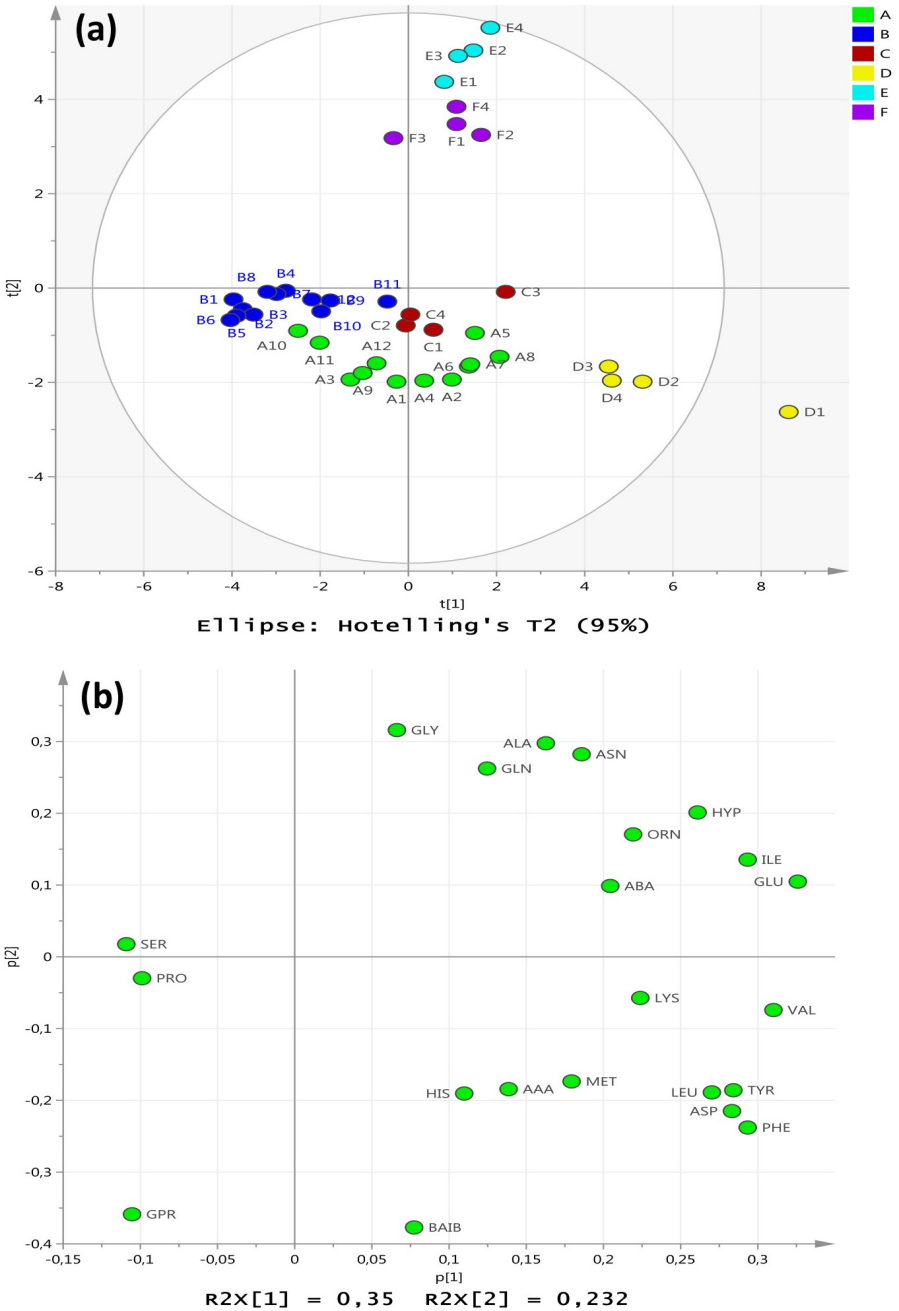
Principle component analysis of *S*. *aureus* cytoplasmic amino acids following growth under the differing experimental regimes. (**a**) PCA scores from the data were assessed for (A) control cultures grown under ideal conditions at pH7 and 37°C with no added NaCl; (B) centroid cultures grown at pH 7 and 37°C with 2.5% NaCl, and the four experimental groups: (C) 35°C and pH6 with no added NaCl; (D) 35°C and pH8 with no added NaCl; (E) 35°C and pH6 with 5% NaCl added; (F) 35°C and pH8 with 5% NaCl. (**b**) The PCA loadings p_1_ and p_2_ indicating the contributions of the amino acids to the cluster separations.

Multivariate analysis using Principle components analysis (PCA) was undertaken to further investigate the differences in the cytoplasmic amino acid profiles between the control, centroid and experimental groups. The unit variation (UV) scaled PCA of the amino acids used 96% of the data (R^2^ = 0.85; Q^2^ = 0.49) after 5 components, whereafter the Eigenvector value levelled out. However, the two first components included most of the cross-validation (CV) (i.e. Q^2^ = 0.40). The analysis revealed scores which clustered the sample replicates into their various treatment groups as shown in Fig 1(a). The PCA loadings p_1_ (see Fig 1(b)) indicated that the majority of the amino acids contributed to the cluster separation. This analysis showed that the biological replicates were tightly clustered depending on the environmental conditions for growth, indicating high reproducibility within replicates with characteristic amino acid profile compositions between treatments (Fig 1(a)).

**Fig 2:**
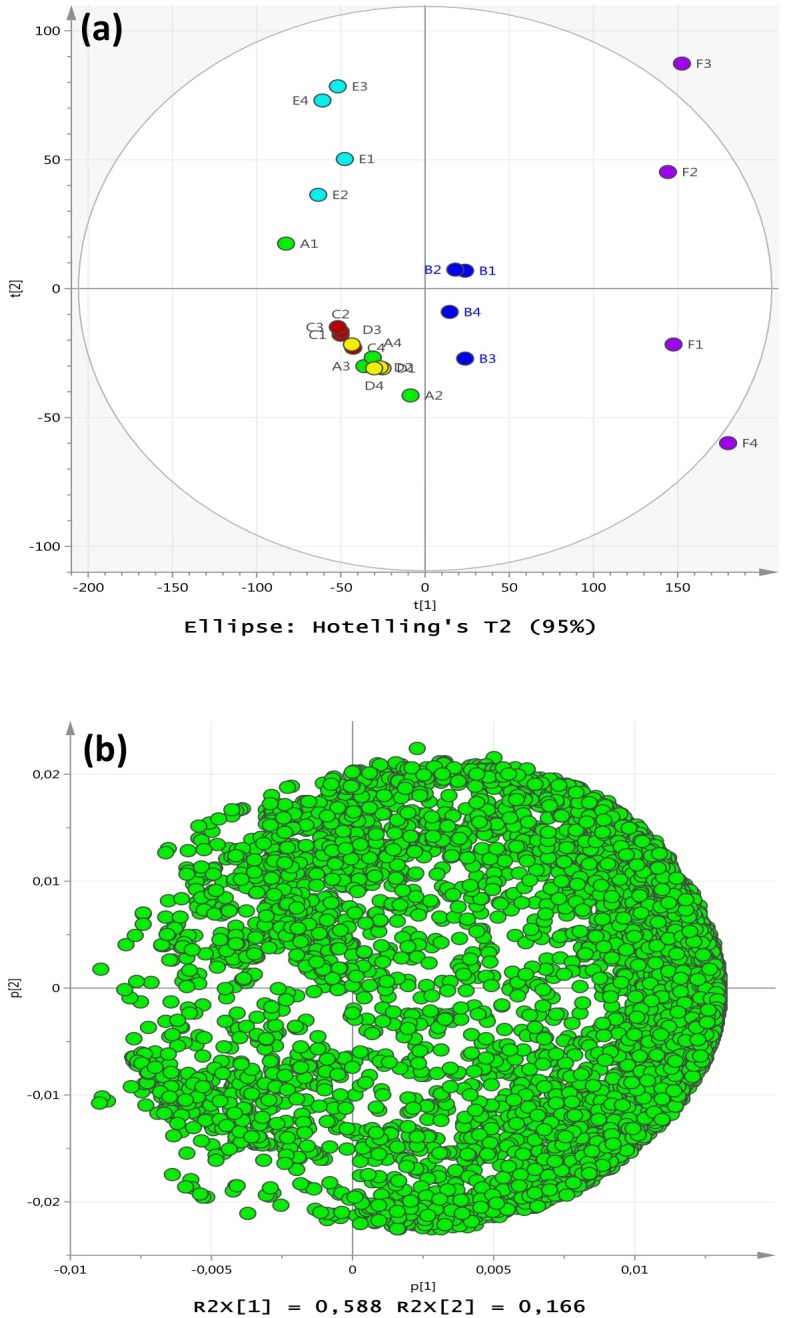
Principle component analysis of *S*. *aureus* proteomic following growth under the differing experimental regimes. (a) PCA scores from the proteomic data were assessed for (A) control cultures grown under ideal conditions at pH7 and 37°C with no added NaCl; (B) centroid cultures grown at pH 7 and 37°C with 2.5% NaCl, and the four experimental groups: (C) 35°C and pH6 with no added NaCl; (D) 35°C and pH8 with no added NaCl; (E) 35°C and pH6 with 5% NaCl added; (F) 35°C and pH8 with 5% NaCl. (b)The PCA loadings p_1_ and p_2_ indicating the contributions of the peptides to the cluster separations.

The cytoplasmic tryptic-digested proteins were analysed by LC-MS/MS for determining protein responses under control, centroid and experimental groups (S1 Data). All the identified peptide components obtained from mass spectrometry were subjected to multivariate analysis using PCA. The model obtained included 8 CV significant components (R^2^ = 0.95 and Q^2^ = 0.84) but most of the explained variance was provided by the first 3 components (R^2^ = 0.83 and Q^2^ = 0.72) where the first component modelled factor variation, and the second and third both modelled factor settings and within replicate differences in parallel. The results revealed that those treatments with added salt were well resolved from each other (groups B, E and F) and well resolved from those treatments with no added NaCl (groups A, C and D) as shown in Fig 2(a). The treatment groups with no added NaCl were very closely positioned on the t1 vs t2 plot indicating that their protein profiles were relatively similar. The positioning of members from group (F) with pH8 and 5% added salt was far resolved from all other treatments with considerable dispersal along t2. The change of conditions to 2.5% added salt (B) represented the smallest change in environmental conditions relative to the control and this group of replicates was positioned closest to the reference control group (A) as well as the other treatments with no added NaCl (C and D). The pH imposed a substantial effect on the response to adding 5% NaCl with excellent separation of the cells from pH6 (group E) compared with the cells at pH 8 (group F). The loading p1 versus p2 scatter plot indicated by density that the first component correlated (on average) to higher peptide abundances, while the p2-vector appeared neutral in that aspect. Thus the treatment (F) at 35°C and pH8 with 5% NaCl appeared to induce the most numbers of increases in peptide levels compared with the other factorial settings (Fig 2(b)).

Initial review of the peptide data revealed that 45 of the ribosomal proteins were detected via the peptide analyses and most were shown to have significant alterations in one or more of the treatment regimes. Peptide sequences and relative statistics information have been provided as supplementary data. This group of ribosomal proteins was thus selected for specific evaluation of cellular responses to altered environmental conditions that mimic a wound site and the results have been summarised by comparison with the control levels in Table 2.

**Table 2:** Ribosomal protein compositions in *S*. *aureus* following growth under the various sets of environmental conditions in comparison to reference control samples (n = 4).

Treatments	(B) Centroid pH7; 37°C; 2.5%NaCl; n = 4	(C) pH6; 35°C; 0%NaC; n = 4	(D) pH8; 35°C; 0%NaCl; n = 4	(E) pH6; 35°C; 5%NaCl; n = 4	(F) pH8; 35°C; 5%NaCl; n = 4
Ribosomal proteins					
L1	Up-regulated	NC	NC	Down-regulated	Up-regulated
L2	Up-regulated	NC	NC	Down-regulated	Up-regulated
L3	NC	NC	NC	Down-regulated	Up-regulated
L4	Up-regulated	NC	NC	Down-regulated	Up-regulated
L5	Up-regulated	NC	NC	NC	Up-regulated
L6	Up-regulated	NC	NC	Down-regulated	Up-regulated
L7/L12	NC	NC	NC	Up-regulated	Up-regulated
L9	Up-regulated	Down-regulated	Down-regulated	Down-regulated	Up-regulated
L10	NC	Up-regulated	NC	Up-regulated	NC
L11	Up-regulated	NC	NC	Down-regulated	Up-regulated
L13	Up-regulated	NC	NC	Down-regulated	Up-regulated
L14	Up-regulated	NC	NC	Down-regulated	Up-regulated
L15	Up-regulated	Down-regulated	NC	Down-regulated	Up-regulated
L17	Up-regulated	NC	NC	Down-regulated	Up-regulated
L18	Up-regulated	NC	NC	Down-regulated	Up-regulated
L19	Up-regulated	NC	NC	Down-regulated	Up-regulated
L20	Up-regulated	NC	NC	Down-regulated	Up-regulated
L21	Up-regulated	NC	NC	Down-regulated	Up-regulated
L22	NC	NC	NC	Down-regulated	Up-regulated
L23	NC	NC	NC	Down-regulated	NC
L25	Up-regulated	NC	NC	Down-regulated	Up-regulated
L27	Up-regulated	NC	NC	Down-regulated	Up-regulated
L28	Up-regulated	NC	NC	NC	NC
L29	Up-regulated	NC	NC	Up-regulated	Up-regulated
L30	Up-regulated	NC	NC	NC	Up-regulated
L31 type B	Up-regulated	NC	NC	NC	Up-regulated
L32	NC	NC	NC	NC	NC
L33	Up-regulated	NC	Down-regulated	NC	Up-regulated
S2	Up-regulated	NC	NC	Down-regulated	Up-regulated
S3	Up-regulated	NC	NC	Down-regulated	Up-regulated
S4	Up-regulated	NC	NC	Down-regulated	Up-regulated
S5	Up-regulated	NC	NC	Down-regulated	Up-regulated
S6	Up-regulated	Down-regulated	NC	NC	Up-regulated
S7	Up-regulated	NC	NC	Down-regulated	Up-regulated
S8	Up-regulated	NC	NC	Down-regulated	Up-regulated
S9	Up-regulated	NC	NC	Down-regulated	Up-regulated
S10	Up-regulated	NC	NC	Down-regulated	Up-regulated
S11	Up-regulated	NC	NC	Down-regulated	Up-regulated
S12	Up-regulated	NC	NC	Down-regulated	Up-regulated
S13	Up-regulated	NC	NC	Down-regulated	Up-regulated
S16	Up-regulated	NC	NC	Down-regulated	Up-regulated
S17	Up-regulated	NC	NC	NC	Up-regulated
S18	NC	NC	NC	Down-regulated	NC
S19	NC	NC	NC	NC	NC
S20	Up-regulated	NC	NC	NC	Up-regulated

Control cultures were grown under ideal conditions at pH7 and 37°C with no added NaCl; (B) centroid cultures grown at pH 7 and 37°C with 2.5% NaCl; (C) 35°C and pH6 with no added NaCl; (D) 35°C and pH8 with no added NaCl; (E) 35°C and pH6 with 5% NaCl added; (F) 35°C and pH8 with 5% NaCl. Significant change at P<0.05, NC = No Change.

When the cells were exposed to an additional 2.5% NaCl at pH7 and 37°C (B), 37 out of 45 ribosomal proteins displayed a significant up-regulation in concentration relative to the control samples (A). Those that did not increase included L3, L7/L12, L10, L22, L23, L32, S18 and S19. The addition of 5% NaCl at 35°C at pH8 (F) also resulted in elevated levels of 39 out of 45 ribosomal proteins compared with the control but no changes were observed for L10, L23, L28, L32 and S18 and S19. In contrast however, addition of the 5% added NaCl at 35°C and pH6 (E), resulted in diminished levels of 32 out of 45 ribosomal proteins where L5, L28, L30, L31 type B, L32, L33, S6, S17, S19 and S20 did not alter in concentration, but L7/L12, L10 and L29 were up-regulated in concentration compared with the control. When the cells were subjected to the 35°C regimes without added NaCl, no general responses were observed for the majority group of ribosomal proteins. However, at pH6 and 35°C (C), a significant increase was observed in L10 with concurrent reductions in L9, L15 and S6, whereas at pH8 and 35°C (D) there were reductions in L9 and L33.

The ribosomal proteins that did not alter in cytoplasmic composition for the various treatments have been summarised in Table 3 where it was apparent that L32 was the only ribosomal protein that did not change in cytoplasmic concentrations across all experiments (B-F). The L23 ribosomal protein did not increase in the two treatments that resulted in a general increase in the other ribosomal proteins (B and F). L9 was the only ribosomal protein that was reduced in cytoplasmic concentration in both treatments where there was no general ribosomal response (C and D, Table 3).

**Table 3:** Summary of ribosomal protein alterations in the *S*. *aureus* cytoplasmic extracts following growth under the various sets of environmental conditions in comparison with the reference control samples.

Growth conditions	Ribosomal proteins identified which did not alter in cytoplasmic concentrations whilst the majority of the ribosomal proteins were either up-regulated or down-regulated in cytoplasmic composition relative to the control	Ribosomal proteins that had significant increases or decreases relative to the control in contrast to the responses for the majority of ribosomal proteins
(B) Centroid: pH7; 37°C; 2.5% NaCl; n = 4	S18: S19; L3; L7/L12; L10; L22; **L23**; **L32**	
	*Majority of ribosomal proteins increased*	
(C) pH6; 35°C; 0%NaC; n = 4		*Up-regulated*: *L10*
	*No majority ribosomal protein response*	*Down-regulated*: **L9**; L15; S6
(D) pH8; 35°C; 0%NaCl; n = 4		*Up-regulated*:
	*No majority ribosomal protein response*	*Down-regulated*: **L9**; L33
(E) pH6; 35°C; 5%NaCl; n = 4	S6; S17; S19; S20; L5; L28; L30; L31 Type B; **L32**; L33	*Up-regulated*: L7/L12; L10; L29
	*Majority of ribosomal proteins decreased*	*Down-regulated*:
(F) pH8; 35°C; 5%NaCl; n = 4	S18; S19; L10; **L23**; L28 **L32**	
	*Majority of ribosomal proteins increased*	

Control cultures were grown under ideal conditions at pH7 and 37°C with no added NaCl; (B) centroid cultures grown at pH 7 and 37°C with 2.5% NaCl; (C) 35°C and pH6 with no added NaCl; (D) 35°C and pH8 with no added NaCl; (E) 35°C and pH6 with 5% NaCl added; (F) 35°C and pH8 with 5% NaCl

**Fig 3:**
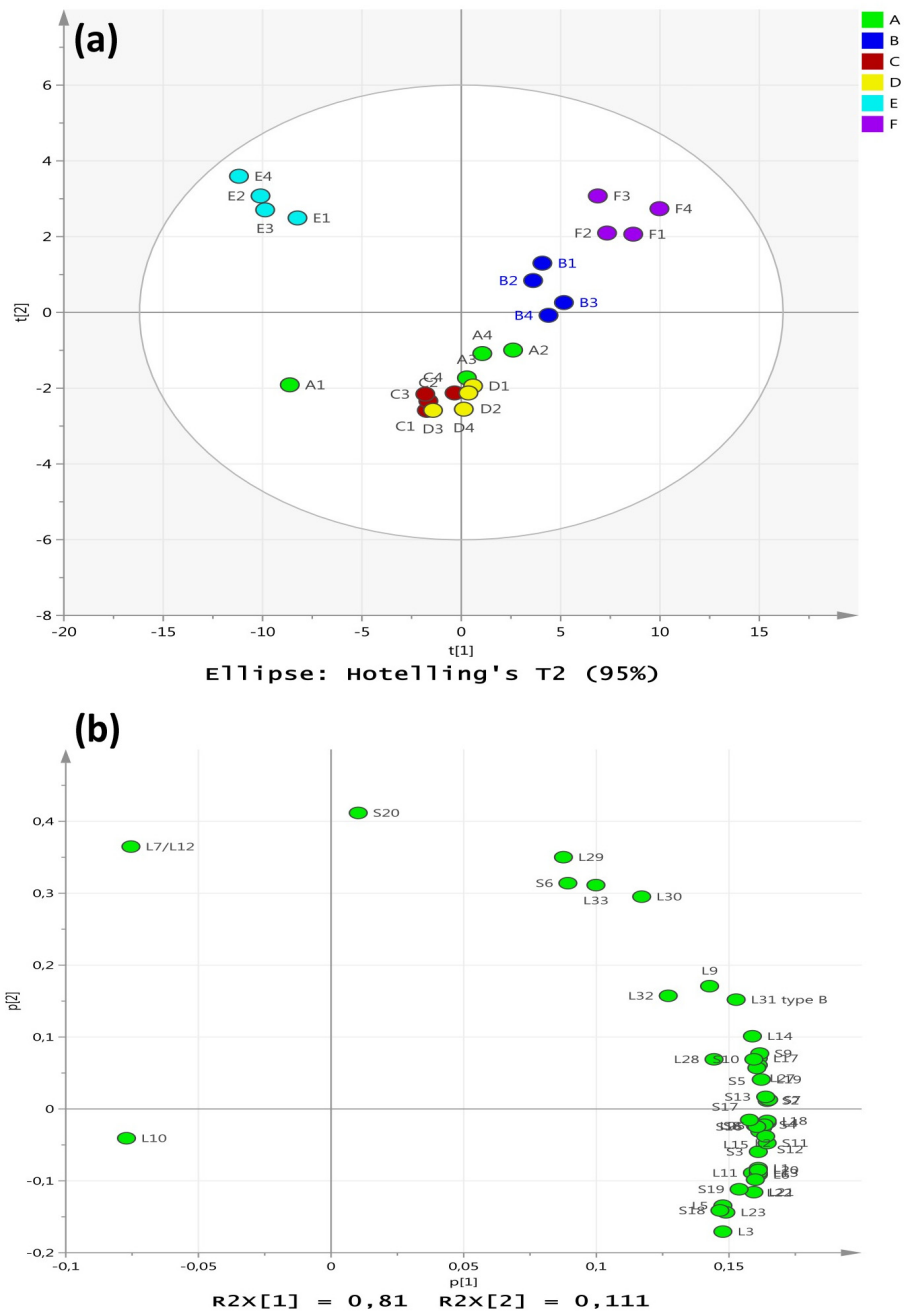
Principle component analysis of *S*. *aureus* ribosomal proteins following growth under the differing experimental regimes. (a) PCA scores from the ribosomal protein data derived from S. aureus cells grown under control, centroid and experimental regimes. The data were assessed for (A) control cultures grown under ideal conditions at pH7 and 37°C with no added NaCl; (B) centroid cultures grown at pH 7 and 37°C with 2.5% NaCl, and the four experimental groups: (C) 35°C and pH6 with no added NaCl; (D) 35°C and pH8 with no added NaCl; (E) 35°C and pH6 with 5% NaCl added; (F) 35°C and pH8 with 5% NaCl. (b) The PCA loadings p_1_ and p_2_ indicating the contributions of the ribosomal proteins to the cluster separations.

The ribosomal protein components assessed in the cytoplasmic extracts from cultures grown under control, centroid and experimental groups were subjected to multivariate analysis using PCA as shown in Fig 3(a). The PCA generated a two component model (R^2^ = 0.92 and Q^2^ = 0.89) indicating solid significance. The clustering of scores indicated that the first component comprised the experimentally instilled factors, while the second component modelled within factor variation (see Fig 3(a)). The control samples (A) were closely aligned with the cells grown at 35°C and pH6 (C) or pH8 (D) with no added NaCl which was consistent with the similar profiles shown in Table 2 as well as the full proteome appraisal shown in Fig 2. The centroid samples (B) were well resolved from the aggregation of control (A) and the (C) and (D) sample sets where the centroid response (B) involved a considerable up-regulation of the majority of the ribosomal proteins compared with the control sample. The two treatments at 35°C with an additional 5% NaCl at pH6 (E) or pH8 (F) were well resolved and had opposing responses where the treatment at pH6 revealed a general downturn in ribosomal proteins whereas those at pH8 showed an increase in the majority of these proteins (Table 2). Thus the treatment (F) at 35°C and pH8 with 5% NaCl appeared to induce the most increases in ribosomal levels compared with the other factorial settings (Fig 2(b)).

## Discussion

The results from this investigation demonstrated that when *S*. *aureus* was grown under a range of environmental conditions representative of the human wound site, the cells adapted by altering their cytoplasmic composition of amino acids and proteins. The substantial and characteristic alterations in proteomic and metabolic profiles observed under each set of environmental conditions provided evidence that major shifts in metabolic homeostasis were generated by the cells to survive these small changes in environmental conditions. Prolonged exposure to extreme changes in temperature or antibiotics has led to the formation of small colony variants on plate cultures [11, 30, 31] and cells with altered morphological characteristics as well as metabolic and proteomic profiles in broth cultures [11, 13, 17]. The changes in environmental conditions in the current study were not extreme, but the cells underwent substantial processes to optimise their growth potential which could be argued to represent distinct phenotypes under each set of conditions. The control and centroid regimes were repeated as three separate sets of four replicates across the experimental period and the results indicated that replication of growth conditions led to reproducible metabolic phenotypes at the time of harvest in regards to cytoplasmic amino acid composition. Alteration of any one or more of the environmental parameters led to a different set of reproducible cytoplasmic profiles of amino acids.

It was also clear that synergistic (and/or antagonistic) interactions occurred between environmental parameters such as temperature and pH, which would differentially govern how the cells would respond to changes in any one specific variable such as salinity. For example, the amino acid responses in treatment (E) for growth under conditions of 5%NaCl at pH6 (35°C) involved a significant increase in total cytoplasmic amino acids relative to the control compared with a reduction observed for treatment (F) under conditions of 5% NaCl at pH8 (35°C, Table 1). The two growth regimes yielded cells which had different cytoplasmic amino acid profiles as shown in Table 1, and Fig 1. Growth regime (E) yielded cells displaying a down-regulation of ribosomal proteins that corresponded to an increase in cytoplasmic amino acids. This may reflect a stock-piling effect in the cytoplasm if protein synthesis was reduced. Conversely, growth regime (F) yielded cells with an upregulated set of ribosomal proteins and a correspondingly lower cytoplasmic level of amino acids compared with group (E). Under conditions of pH 8, there may be a higher demand for new protein synthesis and/or higher rates of protein turnover to survive the added salinity (5%NaCl) reflecting a higher utilisation of amino acids in the cytoplasm. A previous study examined the adaptation of *S*. *aureus* to lung environments and showed that most of ribosomal protein genes were up-regulated with exception of ribosomal protein L9, L33, S21, L7Ae, the putative ribosomal-protein alanine-acetyltransferase SACOL2039 and ribosomal protein L11 methyltransferase gene *prmA* [32]. In a similar way, ribosomal proteins were also up-regulated in the small colony variants of *S*. *aureus* that were isolated from an osteomyelitis patient treated with the antibiotic gentamicin [33]. *Listeria monocytogenes* has also displayed an extensive up-regulation of ribosomal protein genes in response to cold stress and hyperosmotic stress [34], but down-regulation of set of ribosomal proteins in response to chlorine dioxide [35].

The results from the current study also suggested that the responses by the cells to environmental fluctuations depended on the culmination of multiple chemico-physico parameters in the environment. There is an implied capacity by the bacteria to detect and respond to variations in environmental conditions to adjust protein synthesis and metabolic homeostasis. A similar experimental approach was applied to *S*. *lugdunensis* where it was shown that significant alterations in membrane fatty acid composition occurred in response to identical variations in pH, temperature and added NaCl [19]. Further studies using FTIR on *S*. *aureus* under an identical design revealed responses attributable to fatty acids and proteins that were characteristic of the prevailing environmental conditions [20]. On this basis, a hypothesis was proposed whereby the cells continually detect and adjust to the environment via epigenetic mechanisms to produce the most effective phenotype for optimal survival in the current conditions [19, 36]. It is also likely that this would be integrated with the process of bet-hedging where multiple phenotypes co-exist in any population with the most predominant type governed by the prevailing conditions [37]. This could provide a highly effective and efficient mechanism for survival against rapid changes in the environment [38].

The specific alterations in cytoplasmic amino acids may reflect a range of demands for protein synthesis where altered sets of proteins would be required under the different growth conditions. The amino acids also represent activity-snapshots of various biochemical pathways utilised in their synthesis. The precise roles of the altered states of biochemical homeostasis were not obvious at this stage, but relative abundances within the profile were consistent between replicates under a given set of environmental conditions and reproducible across repeat experiments. Glutamic acid, aspartic acid, proline and alanine were the most abundant cytoplasmic components undergoing homeostatic adjustments under different growth regimes but other smaller abundant amino acids such as valine and glycine were also clearly differential in responses between treatments. These results suggested that the reigning amino acid profile in the cytoplasm was not simply a reflection of left over resources from protein synthesis but contributed to ongoing metabolism and nitrogen balance to optimise survival capacity.

The high abundance of glutamic acid may reflect its utilisation in numerous metabolic pathways involved in adjusting to environmental influences. Apart from being able to be readily oxidised for energy, the D-form of this amino acid produced by glutamate racemase is an essential component of the peptidoglycan bacterial cell wall [39]. Cell wall thickness in *S*. *aureus* has been shown to increase in response to exposures to cold stress, penicillin and G vancomycin [11, 13] suggesting that synthesis and protein turnover of the cell wall is an important factor in the response to alterations in environmental conditions. Further to bacterial cell wall synthesis and general protein turnover, glutamic acid is required as a vital source for bacterial integrity as it feeds into metabolic pathways for the production of histidine, glutamine, proline, arginine, glutathione, porphyrin ring and nucleotides via glutamine [40, 41].

Osmoprotectant amino acids such as proline and glycine, as well as glycine-betaine, have been reported to increase in response to elevated salinity [42–44]. Compared with the control conditions in the present study, glycine was significantly increased in all the cells grown with an additional 5% NaCl, which was consistent with the earlier studies. Proline was significantly increased in the cells grown at 37°C and pH7 with the addition of 2.5% NaCl and the cells grown at 35°C at pH6 with the addition of 5% NaCl. However, it was significantly decreased in the cells grown at 35°C and pH8 with the addition of 5% NaCl. These results demonstrated that changing the medium from acidic to alkaline pH reversed the cell response for maintaining proline concentration in the cytoplasm. It has been suggested that proline works as a chaperon molecule, and plays an important role in maintaining protein integrity and enzyme activities [45, 46] as well as acting as an antioxidant [47, 48]. This may explain the significant alterations of proline in this study observed under the various environmental regimes. Amino acids have critical roles in numerous biochemical pathways which may be regulated by responses to environmental conditions that would govern their rate of usage from the cytoplasmic pool.

The PCA analysis of the complete proteomic dataset revealed that the cells grown under control conditions (A) and those grown at 35°C with no added NaCl at either pH6 (C) or pH8 (D) were grouped together in Fig 2(a) reflecting broadly similar cytoplasmic protein compositions. The cells grown with an additional 2.5% NaCl at pH7 and 37°C (B) resulted in a distinctive protein profile that separated this group of samples from the aggregation of groups (A), (C) and (D). The cells grown at 35°C and 5% added NaCl at pH6 (E) and pH8 (F) were well resolved from each other and well separated from cells grown under all other conditions. It was concluded that the challenge of increased salinity in the growth conditions led to considerable alterations in protein composition to enable proliferation of the *S*. *aureus* cells in culture. The loadings shown in Fig 2(b) provided evidence to substantiate this interpretation and the pH had substantial impact on directing the nature of the proteomic response. The extent of the alterations in overall protein composition in the cytoplasm supports the hypothesis that *S*. *aureus* responds to changes in environmental conditions via substantial alterations in the proteome which may be representative of a phenotypic shift. The results from this study also suggested that the bacterium is continually responding to the dynamic environment via modifying the proteome and optimising metabolic homeostasis. This would be an excellent survival advantage and may represent a key attribute in the success of staphylococci as opportunistic pathogens.

The PCA provided a powerful platform for investigations of the different omics arrays. In the present study, the factorial design of experiments provided biological insights on the extent to which the bacteria responded to the combinations of changes in environmental conditions for growth and provided data structures for the comparisons of omics arrays. The amino acid and protein models resulted in distinct clustering patterns for the cells derived from the different growth conditions but these clustering patterns did not fully overlap. This could be explained if the cytoplasmic pool of amino acids were not solely there to service protein synthesis. The amino acids have a broad range of vital roles in metabolism such as being precursors for the synthesis of nucleotides and nucleic acids, the formation of methylene tetra-hydrofolate, formation of complex lipids (e.g. phospholipids) [19] and of course their use as potential energy substrates. This would infer that the cytoplasmic proteome and the amino acid pool do not fully correlate for generating the phenotypic change, a concept that will need to be investigated by dedicated correlation methods in future investigations.

Analysis of the ribosomal proteins showed that the majority of this group of proteins were altered when *S*. *aureus* was exposed to any of the growth regimes involving addition of 2.5% or 5% NaCl. The responses by cells to growth under additional NaCl at pH 7 or 8 involved up-regulation of most of the ribosomal proteins whereas the response at pH 6 involved a down regulation. The ribosomal proteins that did not respond to the additional NaCl have been summarised in Table 3 and indicated that each set of growth conditions instigated a characteristic response that excluded specific ribosomal proteins depending on the pH and temperature. It was also interesting to note that at 35°C and pH 6 with 5% salt added (E), L10 and L29 were up-regulated when all other ribosomal proteins were either down regulated or showed no change. These results suggested that not all of the ribosomal proteins were required to facilitate protein synthesis for growth under certain conditions. It was also evident that some of the ribosomal proteins may have very specific roles which do not involve protein synthesis but were required for facilitating growth and adjusting homeostasis as shown by their independent responses under various growth conditions. It has already been established that some ribosomal proteins are not important in the translation apparatus as some such as S1, S21, S22, S31e, and L25 do not exist in all bacteria [49].It is also known that the deletion of some of these ribosomal genes did not affect the viability of the cells [18, 49–51]. It has been suggested that ribosomal proteins have an extra-ribosomal role as they have been found amongst the surface and secreted proteins of cells [51–53] but the certain roles of these secreted ribosomal proteins remain unclear. It is therefore proposed that certain members of the suite of ribosomal proteins may be secreted to the surface of the cell or into the external environment as a defensive mechanism in response to external challenges from host immune system, antibiotics and changing environmental conditions.

This study showed that the majority of ribosomal proteins were up-regulated in the presence of additional NaCl at pH 7 (B) or pH 8 (F), but there was no ribosomal protein group response observed at pH8 without additional NaCl. The up-regulation in the ribosomal proteins was also observed in *S*. *aureus* when it was exposed to cold stress at 4°C [17]. These alterations in environmental conditions may lead to a higher rate of protein damage and thus a demand to support higher levels of protein turnover. It has also been shown that salinity stress greatly influenced the cell size, the content of cell wall-associated proteins as well as ultimately altering the permeability of the cell membrane via the synthesis of specific proteins that were needed to cope with the conditions [11, 13, 19].

Exposing *S*. *aureus* to variations in the environment for growth instigated significant alterations in cytoplasmic amino acids and proteins with distinctive responses noted for certain ribosomal proteins under various environmental conditions. It was concluded that specific protein and amino acid alterations would provide the basis for the adaptive mechanisms that have led to the evolutionary survival and optimal conditioning of *S*. *aureus* to take advantage of prevailing conditions and infective opportunities. It was evident that unique and systematic processes were required to survive small variable combinations of environmental conditions to maintain an optimal status of metabolism. It was concluded that these changes in cytoplasmic amino acids and proteome were fundamental as an adaptation process for responding to changes in environmental conditions.

## Supporting Information

S1 Data The peptide sequences and relative abundance information for each ribosomal protein under the different sets of conditions. (XLS)
